# Joint EANM/SNMMI procedure guideline for the use of ^177^Lu-labeled PSMA-targeted radioligand-therapy (^177^Lu-PSMA-RLT)

**DOI:** 10.1007/s00259-023-06255-8

**Published:** 2023-05-29

**Authors:** Clemens Kratochwil, Wolfgang P. Fendler, Matthias Eiber, Michael S. Hofman, Louise Emmett, Jeremie Calais, Joseph R. Osborne, Amir Iravani, Phillip Koo, Liza Lindenberg, Richard P. Baum, Murat Fani Bozkurt, Roberto C. Delgado Bolton, Samer Ezziddin, Flavio Forrer, Rodney J. Hicks, Thomas A. Hope, Levent Kabasakal, Mark Konijnenberg, Klaus Kopka, Michael Lassmann, Felix M. Mottaghy, Wim J. G. Oyen, Kambiz Rahbar, Heiko Schoder, Irene Virgolini, Lisa Bodei, Stefano Fanti, Uwe Haberkorn, Ken Hermann

**Affiliations:** 1 Department of Nuclear Medicine, University Hospital Heidelberg, Heidelberg, Germany; 2 Department of Nuclear Medicine, University of Duisburg-Essen and German Cancer Consortium (DKTK)-University Hospital Essen, 45147 Essen, Germany; 3 Department of Nuclear Medicine, Klinikum Rechts Der Isar, Technical University Munich (TUM), 81675 Munich, Germany; 4 Prostate Cancer Theranostics and Imaging Centre of Excellence, Molecular Imaging and Therapeutic Nuclear Medicine, Peter MacCallum Cancer Centre, Melbourne, VIC Australia; 5 Department of Oncology, Sir Peter MacCallum, University of Melbourne, Melbourne, VIC Australia; 6 Department of Theranostics and Nuclear Medicine, St Vincent’s Hospital Sydney, Darlinghurst, Australia; 7 Ahmanson Translational Theranostics Division, Department of Molecular and Medical Pharmacology, David Geffen School of Medicine at UCLA, University of California Los Angeles, Los Angeles, CA USA; 8 Department of Radiology, Weill Cornell Medicine, New York, NY 10021 USA; 9 Department of Radiology, University of Washington School of Medicine, Seattle, WA USA; 10 Division of Diagnostic Imaging, Banner MD Anderson Cancer Center, Gilbert, AZ USA; 11 Molecular Imaging Branch, Center for Cancer Research, National Cancer Institute, National Institutes of Health, Bethesda, MD USA; 12 F. Edward Hebert School of Medicine, Uniformed Services University, Bethesda, MD USA; 13 Curanosticum Wiesbaden-Frankfurt, Center for Advanced Radiomolecular Precision Oncology, Wiesbaden, Germany; 14 Hacettepe University Faculty of Medicine, Department of Nuclear Medicine, Ankara, Turkey; 15 Department of Diagnostic Imaging (Radiology) and Nuclear Medicine, University Hospital San Pedro and Centre for Biomedical Research of La Rioja (CIBIR), Logroño (La Rioja), Spain; 16 Department of Nuclear Medicine, Saarland University Medical Center, Homburg, Germany; 17 Department of Radiology and Nuclear Medicine, Kantonsspital St. Gallen, St. Gallen, Switzerland; 18 The University of Melbourne Department of Medicine, St Vincent’s Hospital, Melbourne, Australia; 19 Department of Radiology and Biomedical Imaging / Helen Diller Family Comprehensive Cancer Center, University of California San Francisco, San Francisco, CA USA; 20 Department of Nuclear Medicine, Cerrahpasa Medical Faculty, Istanbul University- Cerrahpasa, Istanbul, Turkey; 21 Radiology & Nuclear Medicine Department, Erasmus MC, Rotterdam, The Netherlands; 22 Helmholtz-Zentrum Dresden-Rossendorf (HZDR), Institute of Radiopharmaceutical Cancer Research, Dresden, Germany; 23 Technical University Dresden, School of Science, Faculty of Chemistry and Food Chemistry; German Cancer Consortium (DKTK), Partner Site Dresden, Dresden, Germany; 24 National Center for Tumor Diseases (NCT) Dresden, University Hospital Carl Gustav Carus, Dresden, Germany; 25 Department of Nuclear Medicine, University Hospital Würzburg, Würzburg, Germany; 26 Department of Nuclear Medicine, RWTH Aachen University Medical Faculty, Aachen, Germany; 27 Department of Radiology and Nuclear Medicine, Maastricht University Medical Center (MUMC+), Maastricht, The Netherlands; 28 Department of Biomedical Sciences, Humanitas University, and Humanitas Clinical and Research Centre, Department of Nuclear Medicine, Milan, Italy; 29 Department of Radiology and Nuclear Medicine, Rijnstate Hospital, Arnhem, the Netherlands; 30 Department of Radiology and Nuclear Medicine, Radboud University Medical Centre, Nijmegen, The Netherlands; 31 Department of Nuclear Medicine, University Hospital Muenster, Muenster, Germany; 32 Department of Radiology, Molecular Imaging and Therapy Service, Memorial Sloan Kettering Cancer Center, New York, NY USA; 33 Department of Nuclear Medicine, Medical University Innsbruck, Innsbruck, Austria; 34 Division of Nuclear Medicine, IRCCS Azienda Ospedaliero-Universitaria Di Bologna, Bologna, Italy

**Keywords:** PSMA, EANM/SNMMI, [^177^Lu]Lu-PSMA-617

## Abstract

Prostate-specific membrane antigen (PSMA) is expressed by the majority of clinically significant prostate adenocarcinomas, and patients with target-positive disease can easily be identified by PSMA PET imaging. Promising results with PSMA-targeted radiopharmaceutical therapy have already been obtained in early-phase studies using various combinations of targeting molecules and radiolabels. Definitive evidence of the safety and efficacy of [^177^Lu]Lu-PSMA-617 in combination with standard-of-care has been demonstrated in patients with metastatic castration-resistant prostate cancer, whose disease had progressed after or during at least one taxane regimen and at least one novel androgen-axis drug. Preliminary data suggest that ^177^Lu-PSMA-radioligand therapy (RLT) also has high potential in additional clinical situations. Hence, the radiopharmaceuticals [^177^Lu]Lu-PSMA-617 and [^177^Lu]Lu-PSMA-I&T are currently being evaluated in ongoing phase 3 trials. The purpose of this guideline is to assist nuclear medicine personnel, to select patients with highest potential to benefit from ^177^Lu-PSMA-RLT, to perform the procedure in accordance with current best practice, and to prepare for possible side effects and their clinical management. We also provide expert advice, to identify those clinical situations which may justify the off-label use of [^177^Lu]Lu-PSMA-617 or other emerging ligands on an individual patient basis.

**Supplementary Information:**

The online version contains supplementary material available at 10.1007/s00259-023-06255-8.

## Preamble

The Society of Nuclear Medicine and Molecular Imaging (SNMMI) is an international scientific and professional organization founded in 1954 to promote the science, technology, and practical application of nuclear medicine. The European Association of Nuclear Medicine (EANM) is a professional non-profit medical association that facilitates communication worldwide between individuals pursuing clinical and research excellence in nuclear medicine. The EANM was founded in 1985. SNMMI and EANM members are physicians, technologists, and scientists specializing in the research and practice of nuclear medicine.

The SNMMI and EANM will periodically define new guidelines for nuclear medicine practice to help advance the science of nuclear medicine and to improve the quality of service to patients throughout the world. Existing practice guidelines will be reviewed for revision or renewal, as appropriate, on their fifth anniversary or sooner, if indicated.

Each practice guideline, representing a policy statement by the SNMMI/EANM, has undergone a thorough consensus process in which it has been subjected to extensive review. The SNMMI and EANM recognize that the safe and effective use of diagnostic nuclear medicine imaging requires specific training, skills, and techniques, as described in each document. Reproduction or modification of the published practice guideline by those entities not providing these services is not authorized.

These guidelines are an educational tool designed to assist practitioners in providing appropriate care for patients. They are not inflexible rules or requirements of practice and are not intended, nor should they be used, to establish a legal standard of care. For these reasons and those set forth below, both the SNMMI and the EANM caution against the use of these guidelines in litigation in which the clinical decisions of a practitioner are called into question.

The ultimate judgment regarding the propriety of any specific procedure or course of action must be made by the physician or medical physicist in light of all the circumstances presented. Thus, there is no implication that an approach differing from the guidelines, standing alone, is below the standard of care. To the contrary, a conscientious practitioner may responsibly adopt a course of action different from that set forth in the guidelines when, in the reasonable judgment of the practitioner, such course of action is indicated by the condition of the patient, limitations of available resources, or advances in knowledge or technology subsequent to publication of the guidelines.

The practice of medicine includes both the art and the science of the prevention, diagnosis, alleviation, and treatment of disease. The variety and complexity of human conditions make it impossible to always reach the most appropriate diagnosis or to predict with certainty a particular response to treatment.

Therefore, it should be recognized that adherence to these guidelines will not ensure an accurate diagnosis or a successful outcome. All that should be expected is that the practitioner will follow a reasonable course of action based on current knowledge, available resources, and the needs of the patient to deliver effective and safe medical care. The sole purpose of these guidelines is to assist practitioners in achieving this objective.

## Introduction

Due to its overexpression in most clinically significant prostate adenocarcinomas, the prostate-specific membrane antigen (PSMA) is an important receptor for molecular targeted imaging and therapy. [^177^Lu]Lu-PSMA-617 proved efficacy in a first phase 3 pivotal trial in patients post taxane and one novel-generation androgen-axis targeting drug by demonstrating superiority compared to the standard-of-care (usually a second novel-generation androgen-axis drug) for the co-primary endpoints of progression-free survival and overall survival and secondary endpoints, e.g., quality of life [1]. Consequently, the label obtained from the U.S. Food and Drug Administration (FDA) in March 2022 and the European Medicines Agency (EMA) in December 2022 only differ in detail and [^177^Lu]Lu-PSMA-617 will most likely soon become a mainstay of radiopharmaceutical therapy.

Promising anti-tumor-activity has also been reported for the ligand [^177^Lu]Lu-PSMA-I&T. Academically driven studies and retrospective analyses reported that the pharmacokinetics and dosimetry of [^177^Lu]Lu-PSMA-I&T and [^177^Lu]Lu-PSMA-617 are similar [2]. Likewise, protocols of the respective phase 3 trials that evaluate [^177^Lu]Lu-PSMA-617 (NCT04689828) and [^177^Lu]Lu-PSMA-I&T (NCT04647526) in the taxane-naïve setting share a common design. Thus, currently, it appears reasonable to consider both compounds broadly equivalent for the purpose of this ^177^Lu-PSMA-RLT procedure guideline. However, this guideline recognizes that the level of evidence available for [^177^Lu]Lu-PSMA-I&T is often inferior compared to [^177^Lu]Lu-PSMA-617, but expects that this will be updated as novel phase 3 evidence becomes available.

In addition to [^177^Lu]Lu-PSMA-617 and [^177^Lu]Lu-PSMA-I&T, various PSMA-targeted radiopharmaceuticals based on different ligands or radionuclides are currently under clinical evaluation, e.g., [^177^Lu]Lu-J591 [3], [^177^Lu]Lu-DOTA-rosopatamab (NCT04876651), [^177^Lu]Lu-rhPSMA-10.1 (NCT05413850), [^225^Ac]Ac-PSMA-617 (NCT04597411), [^225^Ac]Ac-PSMA-I&T (NCT05219500), [^225^Ac]Ac-J591 (NCT03276572), [^161^ Tb]Tb-PSMA-I&T (NCT05521412), [^131^I]I-1095 (NCT03939689), [^227^Th]Th-BAY2315497 (NCT03724747), and [^67^Cu]Cu-SAR-bisPSMA (NCT04868604). It is not our objective to perform a rating of the clinical potential of these approaches since the available clinical evidence does not yet allow final conclusions. Due to their different pharmacokinetics or radiation characteristics, these radiopharmaceuticals are not directly comparable and cannot be incorporated into this generic guideline for ^177^Lu-PSMA-RLT.

## Purpose

The purpose of this guideline is to assist nuclear medicine practitioners in the following:

1. Identifying suitable candidates to receive ^177^Lu-PSMA-RLT
2. Performing the treatment procedures
3. Understanding the consequences of therapy—i.e., clinical follow-up, management of possible side-effects, response assessment

## Definitions

1. ^177^Lu: ^177^Lutetium is a medium-energy β-emitter. The electron energies (including β-particles and internal conversion electrons) are mean/max 147 keV/497 keV and correspond to ranges of approx. 0.28 mm/1.8 mm in soft tissue. Its physical half-life is 6.65 days. Gamma co-emissions at 208 keV (emission probability 10.4%) and 113 keV (6.2%) enables detection of contaminations and scintigraphy [4, 5].
2. PSMA: It is a transmembrane enzyme with a large extra-cellular domain that is significantly over-expressed on the cell surface of approx. 85% (range 60–100% depending on the definition of *PSMA-positive*) of prostate adenocarcinomas [6, 7]. With the exception of heterogeneous PSMA expression in neuroendocrine dedifferentiated prostate cancers [8], it positively correlates with grading, i.e., more aggressive tumors tend to have higher expression [9, 10]. Ligand-induced internalization results in partial endosomal trapping of radiolabeled PSMA-ligands [11, 12].
3. RLT: In this guideline, the term *radioligand therapy* (RLT) is used for the low-molecular-weight (< 10 kDa), Glu-urea-based, PSMA-targeting ligands PSMA-617 and PSMA-I&T, whenever the commentary is generic.
4. CRPC: Briefly, castration-resistant prostate cancer, either metastatic (m-prefix) or non-metastatic (nm-prefix) by imaging, is defined by two consecutive PSA progressions (min. 2 weeks apart) to a 25% increase over nadir or appearance of new lesions on imaging, despite hormonal manipulation leading to testosterone serum levels < 50 ng/dl (< 1.7 nmol/l) [13].

## Level of evidence/strength of recommendations

FDA or EMA labels typically rely solely on the protocols and investigator brochures of a few randomized clinical trials (RCTs) [1, 14–17]. Being based on highly standardized inclusion criteria, RCTs do not necessarily reflect typical real-world patients. Prostate cancer patients are often elderly and affected by several comorbidities that would preclude eligibility for clinical trial involvement. Consequently, the results of RCTs may not be generalizable to their individual clinical situation. To enable recommendations regarding clinical situations where final evidence from prospective RCTs does not exist, a review of the literature was done in December 2021 by searching PubMed.gov for the terms “(PSMA) AND (Lu-177 OR 177Lu OR Lutetium-177) AND (therapy OR theranostics OR dosimetry)” and checking results for appropriateness. A similar search strategy and statistical meta-analysis of the identified studies was performed recently [18, 19]. Clinical experience with PSMA-617 was reported in 53 non-randomized prospective studies and retrospective analyses reflecting > 3600 patients even if some overlap of patient cohorts is considered likely [20–72]; PSMA-I&T was assessed in 11 retrospective analyses reflecting approx. 600 patients [2, 73–82]. ClinicalTrials.gov identified ongoing phase 3 trials, considered appropriate to amend important novel information within a foreseeable time. A tabular summary of the studies contributing clinical experience regarding safety and anti-tumor-activity, sorted by PSMA-617 vs. PSMA-I&T and retrospective vs. prospective, is provided in the Annex Tables 1, 2 and 3.

If clinical evidence is available from multicenter RCTs with low risk of bias and confounding, a recommendation is classified as “strong” and should apply to most patients in the particular clinical situation. Meta-analysis and systematic reviews of case control or cohort studies or RCTs with moderate risk of bias lead to moderate-strong recommendations. A clinical benefit appears reasonable for subgroups of patients, but individual risk factors must be considered, respectively. This guideline classifies recommendations as “weak” if they are based on case control or cohort studies (higher risk of confounding or bias than RCTs) or studies that only indirectly demonstrate causal relationships. Even case report and expert opinion (based on personal clinical observation) are considered, if they are addressing clinical important questions. However, in situations with only low quality of evidence available, the best action will likely depend on patient’s individual circumstances.

## Indications

1. Patients with PSMA-positive mCRPC, who progressed under at least one novel androgen-axis drug (e.g., enzalutamide or abiraterone) and at least one taxane regimen (and are unfit for or refuse a second taxane regimen). Strong recommendation according to highest level of evidence: For this setting, the international phase-3 RCT VISION demonstrated superiority of [^177^Lu]Lu-PSMA-617 over the best standard-of-care (defined by physician’s choice) regarding safety, efficacy, and quality-of-life [1].
2. Patients with PSMA-positive mCRPC who progressed under at least one novel androgen-axis drug (e.g., enzalutamide or abiraterone) and docetaxel, but would still be possible candidates to receive cabazitaxel: strong recommendation based on a high level of evidence—for this setting, the 11 center phase-2b RCT TheraP demonstrated higher response rates (biochemical, imaging), longer progression free survival at an equal median overall survival, an increased number of long-term responders at 12 months, better patient-reported outcome in multiple domains, and a reduced number of grade 3/4 toxicities of [^177^Lu]Lu-PSMA-617 compared to cabazitaxel [14].
3. Patients with PSMA-positive but taxane-naïve mCRPC who progressed under at least one novel androgen axis drug (e.g., enzalutamide or abiraterone).

The benefit of ^177^Lu-PSMA-RLT appears reasonable, and it may be more tolerable than docetaxel for patients with individual contra-indications against docetaxel (moderate strong recommendation for this subgroup of patients). For patients in good general condition who are likely to tolerate docetaxel, the balance between possible benefits and therapy-related risks is still uncertain, and ^177^Lu-PSMA-RLT cannot be recommended.

One single-center RCT demonstrated equality in PSA response rate, progression-free survival, or grade 3/4 adverse events between [^177^Lu]Lu-PSMA-617 and docetaxel, but definite conclusions are constrained by low patient numbers (*n* = 20 in both groups). However, a quality-of-life questionnaire favored [^177^Lu]Lu-PSMA-617 [15]. Single-arm retrospective analyses reported favorable tolerability and improved response rates of ^177^Lu-PSMA-RLT in chemotherapy-naïve versus post-taxane patients [20, 73]. However, these single-arm studies did not directly compare ^177^Lu-PSMA versus docetaxel. Consequently, the level of evidence for this setting is still weak.

Currently, [^177^Lu]Lu-PSMA-617 and [^177^Lu]Lu-PSMA-I&T are subject of high quality RCTs evaluating the impact of ^177^Lu-PSMA-RLT in this stage of disease (Annex Table 4). At present, both radiopharmaceuticals are lacking sufficient clinical evidence and no recommendation for one particular ligand can be given.

4. Currently, various clinical situations are being evaluated in ongoing phase 2/3 RCTs (Annex Table 4) and physicians are encouraged to refer patients to RCTs whenever available. If no option to participate in an RCT exists and alternative treatment options have been exhausted or are contra-indicated, it is reasonable and ethically warranted (Article 37 of Helsinki Declaration) to offer ^177^Lu-PSMA-RLT on an individual patient basis or in a compassionate care setting; but national regulations have to be considered.

## Contra-indications

A. Absolute: As ^177^Lu-PSMA-RLT is indicated for life-threatening, malignant disease, it is not reasonable to define absolute contra-indications. In general, the chances to improve should outweigh the risks of harming a patient. Therefore, the indication to treat patients with highgrade myelosuppression should be established with caution, and infrastructure to adequately deal with complications should be available.
B. Relative contra-indications: A brief summary of factors that are typically considered as relative contra-indications are provided in Table 1.

**Table 1:** Relative contra-indications against ^177^Lu-PSMA-RLT

Relative contra-indication	Comment
Life expectancy < 3 months	Except the main purpose is palliative (treatment of tumor-related symptoms)
ECOG ≥ 3	High radiation burden to caregivers and relatives, while most likely only prolonging suffering but not quality-life-time
Unmanageable urinary incontinence	A urinary catheter alone is no contra-indication; it should eventually be even considered to improve radiation protection
Acute urinary tract obstruction or hydronephrosis	Patients with diagnosed or risk of urinary retention, [^99m^Tc]Tc-MAG3 or -DTPA renal scintigraphy should be considered a baseline exam [83]
Unmanageable psychiatric comorbidities	Patient unable to be isolated on a nuclear medicine therapy unit (if requested by local radiation protection regulations)
Other severe (e.g., cardiovascular) comorbidities	e.g., patient must be able to tolerate increased hydration
Progressive deterioration of organ function/risk of multiorgan failure	e.g., GFR < 30 ml/min, creatinine > twofold ULN, liver enzymes > fivefold ULN
Acute infections	–
Myelosuppression^a^	WBC < 2.5/nl ANC < 1.5/nl PLT < 75/nl

^a^In selected cases, it can be reasonable to treat more compromised patients (e.g., “superscan” patients) because improvement of tumor-related baseline cytopenia has been reported several times (weak recommendation, best-available-evidence are case reports and expert opinion, because these patients have a priori been excluded from phase 3 recruitment); but then close monitoring of blood cell count is mandatory

*WBC* total white blood cell-count, *ANC* absolute neutrophil count, *PLT* platelets, *ULN* upper limit of normal, *ECOG* Eastern Cooperative Oncology Group Performance Status
C. Precautions for use:

1. In the VISION trial, combinations with standard-of-care therapy for prostate cancer (analgesics, ADT, NAADs, osteo-protection, and focused radiation therapy) were well-tolerated. No studies regarding the interaction with other medicinal products have been performed.
2. Due to their well-known effects on red-marrow suppression, large-field external beam radiotherapy, chemotherapy, or treatment with radioactive bone-seekers should be discontinued for at least 4 weeks.
3. Clinical experience for patients with moderately reduced kidney function (GFR 30–50 ml/min) is still limited. In patients that started ^177^Lu-PSMA-RLT with reduced baseline kidney function, renal impairment with probabilities contributable to the standard risk factors for chronic kidney disease alone was reported [21, 74]. However, GFR should carefully be monitored from cycle to cycle and ^177^Lu-PSMA-RLT should be discontinued if eGFR declines to < 30 ml/min).
4. Patients with reduced kidney function (delayed blood clearance), extensive previous chemotherapy, or history of prolonged hematological toxicity (both indicators of potentially reduced red-marrow reserve) may be more prone to develop higher grade myelotoxicity.
5. Without specific evidence for ^177^Lu-PSMA-RLT, but according to general knowledge of radiopharmaceuticals: Due to the pro-mutagenic characteristics of any radioactive drug, sufficient contraception is needed during and for 3 months after therapy. Eventually related to the cumulative life dose, the risk to develop irreversible infertility must be considered. The occurrence of therapy-associated myelodysplastic syndrome and secondary leukemia may be increased, particularly in patients with previous extensive exposure to conventional chemotherapy, radio-therapy, or other radiopharmaceutical therapies. These late side effects appear with a delay of several years and are probably not relevant in the setting of mCRPC (even in long-term survivors, they have not been reported), but might be considered if ^177^Lu-PSMA-RLT is used in earlier stage patients.

## Factors associated with survival

Several known prognostic factors (i.e., factors associated with patient outcome, regardless of the specific therapy applied) in mCRPC are also associated with lack of response or short durability of benefit from PSMA-RLT. These prognostic markers can be used by physicians to identify more vulnerable patients. However, due to also being negatively associated with response to several other treatment options, they cannot be used for tailoring therapy [1, 84–86].

Prognostic factors associated with poor treatment outcome:

1. Clinical patient/tumor characteristics: Age < 65, ECOG ≥ 2, symptomatic patients, high Gleason score, short response to hormonal intervention (ADT, NAAD).
2. Imaging findings: Presence of visceral metastases, presence of bone-metastases vs. LN-metastases only, extent of bone metastases, total tumor volume, high [^18^F]FDG-uptake.
3. Lab tests: High PSA, short PSA doubling time, high LDH, high CRP, high ALP, low Hb.

A few predictive factors (i.e., factors associated with clinical outcome of a patient undergoing a specific therapy) have also been identified. These can help stratifying candidate patients to select the most promising subjects for ^177^Lu-PSMA-RLT and, on the contrary, to identify those for whom ^177^Lu-PSMA-RLT may not be the best treatment option [22, 23, 85, 87–90].

Predictive factors associated with sub-standard response to ^177^Lu-PSMA-RLT:

1. Low tumor uptake of radiolabeled PSMA-ligands; also expressed as low tumor absorbed dose in patient-specific dosimetry, low SUVs in PSMA-PET, low tumor/liver-ratio, or low tumor/parotid-ratio in PSMA-SPECT/scintigraphy.
2. Presence of viable ([^18^F]FDG-positive) but PSMA-negative tumor lesions.

As PSMA-PET (or PSMA-SPECT) is a strong and relative unique factor for prediction of an individual patient’s response probability to ^177^Lu-PSMA-RLT, its impact on *patient selection* is thoroughly discussed in the related chapter of this guideline.

## Performing the ^177^Lu-PSMA-RLT procedure

### Facility and personnel prerequisites

Radiopharmaceuticals may be received and administered only by authorized persons in designated facilities that are subject to national regulations. Its radioactive material license must cover appropriate activities of ^177^Lu. The facility in which the treatment is administered must have appropriate personnel, radiation safety equipment, procedures available for handling and disposal of waste, handling of contamination, and monitoring personnel for accidental spills and controlling contamination spread. Normally, the request for ^177^Lu-PSMA-RLT will be initiated by an oncologist or urologist, but ideally by an interdisciplinary tumor-board decision. The nuclear medicine specialist is responsible for the ^177^Lu-PSMA-RLT administration, aftercare, follow-up, and their coordination in close liaison with the referring physicians and other physicians involved in managing the patient. The nuclear medicine specialist is obliged to discuss the technical and clinical aspects of treatment with the patient prior to therapy. A long-term follow-up must be ensured to allow an oncological quality control and detection of possible therapy-related late effects. Involved physicians are encouraged to report any suspected adverse reactions via national reporting systems. Since the treatment is currently standardized and involves the administration of a fixed activity, there is no medical necessity to routinely consult a medical physics expert. However, depending on national legislation and regulations, a medical physics expert should be available for consultation.

In addition to the specific demands of nuclear medicine and radiopharmaceutical therapy, the clinical care for prostate cancer patients generally also involves several other specialists; hence, treatment per multidisciplinary team management (e.g., in a dedicated cancer center) is preferred.

### Patient selection

PSMA-targeted therapy will only be effective against tumor lesions with sufficient expression of the target-receptor. The pattern of tumor PSMA expression should be evaluated per molecular imaging (preferred: ^68^ Ga/^18^F-PSMA PET; alternatively: ^99m^Tc-PSMA SPECT/scintigraphy) together with a conventional comparator (e.g., CT, MRI, or bone scan) to rule out a relevant fraction of PSMA-negative lymph-node or visceral metastases or active/lytic bone lesions (strong recommendation) [91, 92].

The stringent exclusion of all FDG/PSMA mismatch patients on the TheraP trial reduced patient eligibility, but provided higher PSA-response rates compared to VISION that omitted [^18^F]FDG-PET/CT evaluation [1, 14]. However, improved overall survival was only proven by VISION, which also recruited patients with small (< 1 cm visceral or < 2.5 cm lymph node) PSMA-negative tumor sites as long as larger lesions demonstrated uptake greater than liver [1, 93]. Thus, simultaneous [^18^F]FDG-PET appears not mandatory for all patients, but may be helpful in cases with several lesions of uncertain tumor viability or suspicion of PSMA negativity. A conversion from PSMA-positive to PSMA-negative phenotype has repeatedly been reported for liver metastases; hence, especially liver metastases may benefit from a complementary evaluation per [^18^F]FDG-PET (weak recommendation, based on case reports and expert opinion).

Inter-lesion tumor heterogeneity is common in prostate cancer, and PSMA-immunostaining of a single lesion’s biopsy is not necessarily representative for the majority of other tumor sites [94, 95]. Thus, immunohistochemistry is not considered an appropriate substitute for PSMA-PET/SPECT.

Tumor and normal organ uptake depends on the tracer used for PSMA imaging and SUVs depend on reconstruction parameters and scanner calibration. Regarding the currently most frequently used ligands ([^68^ Ga]Ga-PSMA-11, [^18^F]DCFPyL, ^18^F-PSMA-1007, ^18^F-rhPSMA-7.3, [^99m^Tc]Tc-PSMA-I&S, [^99m^Tc]Tc-MIP1404), various groups reported that tumor-to-salivary gland or tumor-to-liver ratios can serve as scanner-independent surrogate criteria [96], but in exchange the confounder “reference-organ variability” becomes an issue. Patients with lesions with diameter ≥ 1 cm showing tumor uptake < 0.5-fold of parotid (which approximately equals < 1.0-fold liver-uptake of [^68^ Ga]Ga-PSMA-11 that has been used in VISION) should be excluded from receiving ^177^Lu-PSMA-RLT [97]. However, response probability correlates with absorbed dose, which (moderately) correlates with uptake values in PET [87, 90, 98].

PSMA uptake may also be used to determine the optimal sequence of therapies. Preliminary data imply that clinically relevant anti-tumor activity is most likely observed in patients with > onefold parotid uptake in the majority of lesions (approx. SUV > 10), and only such patients should actively be encouraged to receive PSMA-RLT [90]; for patients with low PSMA uptake or negative sites, alternative options should be prioritized.

Recommendations regarding the use of ^177^Lu-PSMA-RLT beyond its approved indications are challenging. The decision whether a patient is ineligible for a particular alternative treatment is commonly outside the expertise of a nuclear medicine physician alone and should be assessed with the multidisciplinary team. With regard to androgen deprivation therapy (LHRH-analogs/-antagonists and first-generation antiandrogens) and novel androgen-axis drugs (e.g., abiraterone, enzalutamide), the advice of a board-certified urologist, with regard to chemotherapy, and the advice of a board-certified oncologist or a specialized uro-oncologist can be considered sufficient. The decision of an interdisciplinary tumor-board should be preferred. The right of self-determination is a high value in SNMMI and EANM member states, and patients cannot be forced to accept guideline-advised treatment sequences. Nevertheless, patients should be informed that disregarding disease-related guidelines will commonly preclude reimbursement by the public healthcare provider. In any case, it should be documented that the patient has been informed about potential risks and benefits of the alternative treatment options by an expert in the respective field (board-certified uro-/oncologist).

### Radiopharmaceuticals

Both [^177^Lu]Lu-PSMA-617 and [^177^Lu]Lu-PSMA-I&T belong to the group of PSMA inhibitors that are based on the glutamate-urea-lysine targeting moiety. The precursor PSMA-617 is tagged with a DOTA chelator and has a molar mass of 1042 g/mol (lutetium-labeled: 1216 g/mol). PSMA-I&T is tagged with the DOTAGA chelator, and the precursor molar mass is 1498 g/mol. The sterile therapy solution is typically diluted to ≤ 1000 MBq/ml to reduce the activity concentration in case of extravasation. It is a clear, colorless to slightly yellow solution, free from visible particulates with a radiochemical purity of ≥ 97%.

Currently, only [^177^Lu]Lu-PSMA-617 is available as an approved drug in the USA, Canada, UK, and EU (^177^Lu vipivotide tetraxetan, Pluvicto™). The product liability and compliance to Good Manufacturing Practice, according to USP (US Pharmacopeia) or EP (European Pharmacopoeia) standards, is the responsibility of its commercial provider. This formulation should be used for all approved *in-label* applications of [^177^Lu]Lu-PSMA-617.

Whenever ^177^Lu-PSMA-RLT is considered outside the already-approved indications, the aim should be to recruit patients into a formal clinical trial. In this setting, the compliance of the respective radiopharmaceutical to the Investigational Medicinal Product Dossier or the Investigational New Drug application is ensured by the respective sponsor of the trial.

According to article 7 2001/83/EG, in particular situations, drugs can be used without formal approval. National regulations must be considered. Retrospective analyses of “unproven interventions in clinical practice” and some prospectively-performed single-arm studies (Annex, Table 1, 2 and 3) reported safety and efficacy for various formulations of [^177^Lu]Lu-PSMA-617 and [^177^Lu]Lu-PSMA-I&T following site-specific, in-house labeling conditions. Advice for production and QC of such formulations is not in the scope of this guideline and should only be done in centers with an adequately qualified radiopharmacy. Otherwise, we recommend *off-label* use of the approved formulation whenever patients cannot be recruited to clinical trials when it is possible to obtain the drug and full reimbursement.

### Process of treatment procedure

1. Patient data, medical history (i.e., previous treatments for prostate cancer, current medication for prostate cancer, and comorbidities), recent staging exams, and current general clinical condition are collected. ADT should be continued during PSMA-RLT; bisphosphonates or denosumab are permitted. In the post-taxane setting, standard-of-care treatment with novel androgen-receptor targeting agents might be given in parallel [1, 99]. Informed consent (written and 24 h in advance of first cycle depending on national legislation) must be obtained.
2. Lab tests not older than 5 days are obtained in advance of each treatment cycle: blood cell count, creatinine, eGFR, AST/GOT, ALT/GPT, total bilirubin, albumin, AP/ALP, LD/LDH, PSA, and CRP.
3. The ^177^Lu-PSMA radiopharmaceutical solution is stored below 30 °C in its glass vial inside a lead-shielded unit, where the syringe is prepared under aseptic conditions. The activity in the syringe is measured in a dose calibrator (well counter) before and after administration.
4. Sufficient hydration should be ensured by intravenous infusion (e.g., 500–1000 ml of 0.9% saline at a rate of 250 ml/h) starting 30 min in advance and few hours following administration. In compliant patients, oral hydration is also feasible. Patients should be advised to drink a lot of fluids during the next 1–2 weeks.
5. There is no compelling evidence suggesting mandatory co-medication. Prophylactic antiemetic therapy, e.g., ondansetron, is permitted. Corticosteroids before and up to several days after RLT (mandatory in case of cerebral, spinal, or other metastases with risk of painful or obstructive swelling) are allowed per patient’s need and physician’s choice; most experience exists for an average dose of 4 mg dexamethasone given for 5 days [24].
6. A 10-ml saline flush ensures patency of the IV line before administering therapy. ^177^Lu-PSMA-RLT is slowly administered intravenously over > 30 s and followed by a saline flush with at least twice the volume needed for RLT application.
7. To support kidney clearance, the patient is encouraged to increase fluid intake during the first 3–4 days following treatment. In patients with high tumor burden (increased risk of tumor lysis syndrome), allopurinol can be prescribed during the first week following therapy. Per individual cardiovascular condition, it may be necessary to decrease hydration or to apply diuretics. To reduce bladder dose, patients should void frequently during the first 6–10 h.
8. After administration of the radiopharmaceutical, the patient presents a theoretical risk to other people due to external radiation (gamma co-emissions of ^177^Lu) or possible exposure to excreted radioactivity. Depending on national legislation, up to 48–72 h isolation on a radioisotope ward may be necessary to reach the local discharge threshold (µSv/h). Where out-patient therapy is allowed, patients should nevertheless be kept in isolation for approx. 2 h to monitor side effects, ensure sufficient hydration, and complete first urination before release. Patients should be instructed how to minimize exposure to others (e.g., avoiding prolonged close contact with pregnant women or infants).
9. At least one planar post-therapeutic emission scan should be obtained > 2 h p.i. to rule out extravasation and confirm physiological tracer biodistribution/excretion [100] (cf. Dosimetry section).
10. The discharge letter should contain relevant patient data, date of therapy, the administered radionuclide (^177^Lu) and radiopharmaceutical, administered activity, details on necessary aftercare, and next follow-up appointment.
11. In non-compromised patients, the approved treatment regimen for [^177^Lu]Lu-PSMA-617 is 7.4 GBq (200 mCi) per cycle at 6 w (± 1 w) interval for a maximum of 6 cycles. In clinical practice, safety and anti-tumor activity of [^177^Lu]Lu-PSMA-617 and [^177^Lu]Lu-PSMA-I&T have successfully been demonstrated for a range of 6–9.3 GBq per treatment and for treatment intervals of 4–10 weeks [25, 26].

### Follow-up

1. Blood cell counts and creatinine should be checked at least 2–3 weeks after each cycle (anticipated nadir) and 4–6 weeks after the last treatment cycle; the consequences of pathological findings are described in the “Management of side effects” section.
2. PSA follow-up should be obtained at each cycle as well as 4–6 weeks after the end of therapy and interpreted in accordance with the PCWG3 criteria [13]. Due to the possibility of PSA flare-up (due to wash-out effects) or delayed response, PSA response becomes a reliable marker 2–3 weeks after the 2nd cycle [27, 28, 101]. Then every increase of > 25% should potentially be considered resistance and trigger imaging-based restaging [29].
3. Scintigraphy (optionally SPECT) of the lutetium gamma co-emissions, 1–2 days after infusion, can serve as an easily available imaging to follow-up response of PSMA-positive lesions. A medium energy collimator is recommended to image the upper 208-keV photo-peak [4, 100].
4. Optimal modality for imaging-based restaging is PSMA-PET/CT. Where PSMA-PET is not available, PSMA-SPECT or scintigraphy is possible. It is mandatory to include a second modality to allow detection of possible PSMA-negative lesions; this may be a fully diagnostic CT as part of an integrated PET/CT exam or a dedicated diagnostic CT; in selected patients, also a bone-scan or [^18^F]FDG-PET/CT. Frequency and extent of restaging can be adjusted to the reliability of post-treatment scans and PSA response and is usually recommended every 12 weeks and at the end of each series of PSMA-RLT.
5. Assessment of patient-reported well-being (i.e., fatigue, pain, level of activity) is the key parameter for the decision to continue therapy or not.

### Management of side effects

Expected side effects include fatigue, acute hematological and chronic renal, or chronic salivary gland toxicity. Grade ≥ 3 side effects occur in < 10% of patients.

1. If acute hematological toxicity (i.e., anemia, leukopenia or neutropenia, thrombocytopenia) of grades 3 or 4 is demonstrated at interim lab test between two cycles: Next cycle should be postponed by 2 weeks and might then be canceled when no recovery is seen. For neutropenia, the use of growth factors (G-CSF) is permitted until toxicity resolves to grade 1, but there should be at least a 2-week interval between G-CSFs and injection of radioactivity. For anemia, transfusion or erythropoietin may be given as clinically indicated.
2. If acute hematological toxicity of ≥ grade 2 is demonstrated at the scheduled treatment date: Treatment activity is reduced by 20% or postponed.
3. Acute loss of eGFR > 40% but still > 30 ml/min: Treatment activity is reduced by 20%.
4. Non-hematological toxicities (e.g., gastrointestinal toxicity, fatigue, electrolyte, or metabolic abnormalities) of grade 3 or 4: Hold ^177^Lu-PSMA-RLT until recovery to grade 2 or baseline.
5. Skeletal adverse events: Hold ^177^Lu-PSMA-RLT until complication is adequately treated as deemed appropriate by the treating physician (radiation-oncologist or orthopedic surgeon).
6. Any toxicity that is considered unacceptable by the patient, any life-threatening toxicity that does not resolve within 4 weeks, GFR loss to < 30 ml/min, or unexpected liver toxicity (AST or ALT > fivefold upper-limit of normal): Discontinue ^177^Lu-PSMA-RLT.
7. Mild cases of xerostomia can be alleviated with coping water or sprays with artificial salivary supplements or xylitol lozenges; moderate strong recommendation.
8. “Unacceptable” xerostomia may eventually be improved by retrograde sialendoscopy and flushing with steroids (weak recommendation based on a single cohort study [102]). Due to its own side effects and contra-indications, the use of parasympathomimetics (e.g., the FDA and EMA approved pilocarpine) must consider patient’s individual situation because benefits and risks could be closely balanced [103].
9. A flare-up of tumor-related pain can occur during the first week following therapy but in responding patients often improves below baseline after the second week. Initial worsening of pain can be caused by radiation-induced edema, best responding to steroids or non-steroidal anti-inflammatory drugs. In severe cases, opioids can temporarily be used. Once interim lab tests demonstrate PSA response, an attempt should be made to reduce analgesics.

## Dosimetry

According to the dosimetry sub-study of VISION, the radiation absorbed dose to potentially dose-limiting organs in 29 patients was 2.1 (± 0.47) Gy/GBq for lacrimal and 0.63 (± 0.36) Gy/GBq for salivary glands; kidneys were 0.43 (± 0.16) Gy/GBq and red-marrow 0.035 (± 0.02) Gy/GBq after the first treatment cycle [104]. These doses were extrapolated to all 6 cycles (scaled by a one time-point image for the subsequent cycles) to approximate the cumulative doses for kidneys (19 ± 7.3) Gy and red-marrow (1.5 ± 0.9) Gy with acceptable accuracy [105]. The dosimetry results in this sub-study were consistent with the clinically good safety profile of VISION, with low frequency and severity of radiation-induced adverse events to organs at risk over 6 cycles. Consequently, [^177^Lu]Lu-PSMA-617 was approved using fixed standard treatment activities, and patient individual dosimetry is not mandatory for *in-label* use.

Comparable dosimetry estimates have also been obtained in several academically driven dosimetry studies covering various in-house formulations of [^177^Lu]Lu-PSMA-617 (e.g., with different specific activities GBq/mmol) [22, 23, 31, 36, 61, 70, 87–90, 106–122] (Annex Table-5). Similar absorbed doses to organs-at-risk have also been approximated when using [^177^Lu]Lu-PSMA-I&T [75, 81, 98, 123–126] (Annex Table-5). The technical aspects of the respective studies have recently been summarized in a systematic review [127]. A comparative retrospective analysis, evaluating both radiopharmaceuticals with identical methods, confirmed the similarity of [^177^Lu]Lu-PSMA-617 and [^177^Lu]Lu-PSMA-I&T regarding organs at risk [2] but has the limitation of low patient numbers and retrospective matched-pair analysis.

There are different institutional practices regarding post-therapeutic scintigraphy. They are relatively uncommon in some states of the USA. In contrast, according to EC Directive 2013/59/Euratom article 56, at least one post-therapeutic scan can be considered mandatory for therapy verification in most member-states of the EU. However, for standard treatments, a complete dosimetry is optional [128].

As there is significant inter-patient variability, higher treatment activities or more treatment cycles may be possible for several patients under patient individual dosimetry concepts. Recommendations for patient-specific dosimetry that might allow individualized treatment regimens have been provided by the EANM dosimetry committee recently [100]. The clinical status of the patient should be taken into account when considering serial imaging. The ability of the patient to return for additional visits may also be prohibitive if the imaging center is a fair distance away. Novel simplified dosimetry approximation methods, e.g., based on a single posttreatment SPECT/CT scan, have been developed in single-center studies [87, 106]. Whether they can improve clinical outcome by individualized dosimetry-guided treatment regimens still needs confirmation.

## Open points/miscellaneous

1. Benefit of additional therapy cycles or later re-treatment in advanced disease (higher cumulative absorbed dose) is still unclear and needs to be studied.
2. The impact of fractionation is still unclear. Different dosing regimens with shorter or longer intervals, more or less sessions and eventually considering individual prognostic factors (e.g., PSA doubling time), need to be studied—especially in the perspective that ^177^Lu-PSMA-RLT moves into earlier treatment lines.
3. The clinical relevance and potential consequences of the tumor-sink-effect, i.e., (in)variance of normal organ dosimetry at very high tumor load [129, 130], are still unclear and need to be studied.
4. Several drug interventions (e.g., oral glutamate, atropine injections, pre-dosing with unlabeled PSMA ligands) to reduce off-target uptake of PSMA ligands in salivary glands or kidneys have been suggested [103]. Due to the limited evidence available, at this point, no general recommendation can be given. Please pay attention as such interventions may also affect tumor-to-organ ratios that are used for patient selection.
5. It is not clear whether it is needed (and if yes, how) to take prior doses by external-beam radiotherapy (EBRT) or bone-seeking radiopharmaceuticals into account, or conversely, in the case of EBRT after ^177^Lu-PSMA-RLT (e.g., palliative therapy of painful bone lesions).
6. The prognostic or predictive potential of several molecular biomarkers (e.g., genomic instability, index mutations in DNA or RNA (including splicing variants), tumor stroma or immune related factors) have not sufficiently been evaluated.
7. It remains unclear whether response to previous EBRT has prognostic or predictive impact. However, previous treatment with [^223^Ra]RaCl_2_ had no relevant effect on safety and efficacy of a succeeding ^177^Lu-PSMA-RLT [20].

## Liability statement

This guideline summarizes the views of the EANM and SNMMI Committees/Councils for Oncology, Dosimetry, Therapy/Theranostics, PET, Radiation protection, Radiopharmaceutical Sciences and Technologists. It reflects recommendations for which the EANM cannot be held responsible. The recommendations should be taken into context of good practice of nuclear medicine and do not substitute for national and international legal or regulatory provisions.

## Supplementary Information

Below is the link to the electronic supplementary material.

Supplementary file1 (XLSX 15.4 KB) Annex-Table 1: Safety and Efficacy of 177Lu-PSMA-617

Supplementary file2 (XLSX 10.2 KB) Annex-Table 2: Randomized Clinical Trials of 177Lu-PSMA-617

Supplementary file3 (XLSX 11.1 KB) Annex-Table 3: Safety and Efficacy of 177Lu-PSMA-I&T

Supplementary file4 (XLSX 11.4 KB) Annex-Table 4: Ongoing Clinical Trials with 177Lu-PSMA-617 and 177Lu-PSMA-I&T (registered at https://clinicaltrials.gov/ )

Supplementary file5 (XLSX 11.5 KB) Annex-Table 5: Dosimetry studies of 177Lu-PSMA-617 and 177Lu-PSMA-I&T
